# Towards the use of similarity distances to music genre classification: A comparative study

**DOI:** 10.1371/journal.pone.0191417

**Published:** 2018-02-14

**Authors:** Izaro Goienetxea, José María Martínez-Otzeta, Basilio Sierra, Iñigo Mendialdua

**Affiliations:** 1 Department of Computer Science and Artificial Intelligence, University of the Basque Country UPV/EHU, San Sebastián, Spain; 2 Department of Computer Languages and Systems, University of the Basque Country UPV/EHU, San Sebastián, Spain; Instituto Nacional de Medicina Genomica, MEXICO

## Abstract

Music genre classification is a challenging research concept, for which open questions remain regarding classification approach, music piece representation, distances between/within genres, and so on. In this paper an investigation on the classification of generated music pieces is performed, based on the idea that grouping close related known pieces in different sets –or clusters– and then generating in an automatic way a new song which is somehow “inspired” in each set, the new song would be more likely to be classified as belonging to the set which inspired it, based on the same distance used to separate the clusters. Different music pieces representations and distances among pieces are used; obtained results are promising, and indicate the appropriateness of the used approach even in a such a subjective area as music genre classification is.

## Introduction

Automatic music classification is a topic that is getting more and more attention with the development of the multimedia technologies and the growth of available information. It is used in music genre classification, tune family identification or to classify tunes in geographical regions for example, and approaches that use both symbolic information and audio information have been developed [1, 2].

Music genre classification is an important task since genre is a descriptor that is usually used to organize large collections of music, specially in the Internet, where it is often used in search queries. Many different approaches have been developed to identify music genre in audio or symbolic representation, like Support Vector Machines [3, 4], similarity measures of symbolic representation [5], neural networks [6, 7] or deep learning methods [8].

Automatic music generation has interested people for centuries and many different algorithms have been developed since the first steps in automatic music composition, like knowledge based systems, evolutionary and other population-based methods, fractals or statistical models [9].

The developed methods for music generation can be classified in several categories, like stochastic methods, knowledge-based systems and artificial intelligence systems. Stochastic methods involve random variables and are the simplest to generate. Some early examples can be the *Musikalisches Würfelspiel* or musical dice games, like the one published in 1792 that was attributed to Mozart [10].

Knowledge-based systems use series of sets of rules or grammars to guide the composition of melodies, expanding high-level symbols into sequences of symbols [9]. These grammars can be learned from a corpus of a melodies or they can be invented.

Statistical models have been used in computational modelling of several musical style since they are able to capture some musical features that make it possible to generate new musical sequences that reflect an explicit musical style, and they can be learned from a corpus of melodies [11].

In order to use statistical models for coherent music generation the intra-opus problem needs to be considered: the generated piece must contain material that repeats through the piece. Almost all forms of music involve repetition [12], either of pitch sequences or at some more abstract levels, and that repetition gives a sense of meaning to music [13]. Musical cohesion is analyzed in [14], where music is compared to linguistic discourse, and it is concluded that music is composed by semantically related segments, which support the coherence of the piece. Describing the coherence of a piece is currently a scientific challenge, and different approaches have been developed, like the description of acoustic structure, functional structure or semiotic structure. Semiotic structure is the description of segments in a piece using a set of symbols, where each symbol represents a class of similar segments [15].

Music generation methods using a segmental structure extracted from an existing piece have been developed, to generate music in the “style” of the original piece, but with different melodic content, like the method developed by Collins et al [16]. This method discovers the repeated and transposed segment on a polyphonic piece and uses it to guide the generation of a new melody, which has different notes but the same coherence as the original piece.

This paper presents a folk melody classification method, which is based on similarity distances of symbolic representation of music, and which is combined with an automatic generation method. An unsupervised classification of a folk melody corpus is made and the discovered sets are used to generate new melodies, which are then classified into the discovered clusters.

The chosen corpus is a collection of *bertso* melodies. *Bertsolaritza* or *bertsolarism* is the art of singing improvised songs in Basque (bertsos), respecting various melodic and rhyming patterns. It is defined as a sung, rhymed and metered discourse by the book *The Art of Bertsolaritza: Improvised Basque Verse Singing* [17]. There is evidence of bertso singing and written bertso poem samples since the 15th century, and it is a very popular art nowadays in the Basque Country.

Bertsos are sung in many different occasions, like informal lunches with friends, homage ceremonies or competitions and any topic can occur in a bertso. Many bertsolari competitions take place every year in the Basque Country, and every four years the national championship final is held, with around 15000 people in attendance.

The main technical aspects of the bertso are the rhyme, meter and melody, which can be classified into traditional folk melodies (the great majority), modern melodies that coincide with one of the traditional metres and melodies that are specifically composed. Experts say the chosen melody for singing a bertso and the manner in which it is sung can be the key for the communicative success of the bertsolari, since the chosen melody must be able to combine with the created lyrics to transmit what the bertsolari wants to express with the bertso.

This paper is structured as follows; Section ‘related work’ overviews the work that has been done in music classification, Section ‘proposed approach’ describes the approach we propose, Sections ‘experimental setup’ and ‘experimental results’ present the experimental setup designed to test the method and the results obtained, and finally Section ‘conclusions and future works’ presents the conclusions that have been extracted from this work.

## Related work

Several approaches have been used in the literature to deal with music classification for different tasks, like tune family identification or automatic music genre classification. The idea behind many of them is to obtain a representation of the analyzed music and afterwards build a model which would be able to classify the characteristics of the music treated on the approach, namely genre, structure, artist, composer, and so forth.

Automatic music genre classification is a task that has attracted the interest of the music community for more than two decades, and several similarity methods and machine learning techniques have been studied in the literature to deal with it. Kotsifakos et al. [5] deal with music genre classification for symbolic music, and specifically MIDI, by combining the recently proposed novel similarity measure for sequences, SMBGT, with the k-Nearest Neighbor (k-NN) classifier. For all MIDI songs they first extract all of their channels and then transform each channel into a sequence of 2D points, providing information for pitch and duration of their music notes.

Mendel and Ellis [4] present an approach based on support vector machines to classify songs based on global features.

Chai and Vercoe [18] worked on the classification of folk music pieces coming from different countries using monophonic melodies by means of hidden Markov models. In this paper the authors state that “This shows that melodies of folk music do carry some statistical features to distinguish them”.

Bergstra, J et al. [19] present an algorithm based on ADABOOST that predicts musical genre and artist from an audio waveform.

Xu et al. [20] propose effective algorithms to automatically classify and summarize music content. Support vector machines are applied to classify music into pure music and vocal music by learning from training data. Based on calculated features, a clustering algorithm is applied to structure the music content.

Fu et al. [21] deal with music information retrieval (MIR), which addresses the problem of querying and retrieving certain types of music from large music data set.

Pinquier et al. [22] deal with a novel approach to speech/music segmentation. Three original features, entropy modulation, stationary segment duration and number of segments are extracted. They are merged with the classical 4Hz modulation energy.

Zhang et al. [8] propose the use of computational deep learning modules for extracting invariant and discriminative audio representations which can then be used to classify music in different genres.

Sturn [23] argue that an evaluation of system behaviour at the level of the music is required to usefully address the fundamental problems of music genre recognition (MGR), and indeed other tasks of music information retrieval, such as autotagging.

A challenging open question in music classification is which music representation (i.e., audio features) and which machine learning algorithm is appropriate for a specific music classification task. The goal is to find a set of linear mappings from several feature spaces to the semantic space spanned by the class indicator vectors [24]. Valverde-Rebaza et al. [25] present a novel feature vector obtained directly from a description of the musical structure described in MIDI files for music representation.

Recently Febres and Jaffe [26] proposed a new music representation and classification system based on extracting the *Minimal Entropy Description* of polyphonic music files. The Minimal Entropy Description is the sequence of characters forming symbols for which the corresponding entropy is minimal, and this representation is used to compare computer files associated to a score, considering already available parameters such as their symbolic diversity and entropy.

In the work of Lee et al. [27] the bag of words (BoW) representation of modulation spectral analysis of spectral as well as cepstral features are constructed for music genre classification. This is an approach used as well in text classification [28] which can be improved by means of a Singular Value Decomposition approach [29].

Recent success with deep neural network architectures on large-scale datasets has inspired numerous studies in the machine learning community for various pattern recognition and classification tasks such as automatic speech recognition, natural language processing, audio classification and computer vision [30–32]. Music genre classification has been done as well; Rajann et al. [33] show that neural networks are comparable with classic learning models when the data is represented in a rich feature space. Chun and Hong [34] used a BP neural network (BPNN) music classification method.

In this paper, Basque Folk music is used to perform the experiments; Bassiou et al. dealed with Greek folk music genre classification [35]. Hillewaere et al. worked on automatic classification of dances using the *Dance-9* corpus [36].

## Proposed approach

**Fig 1:**
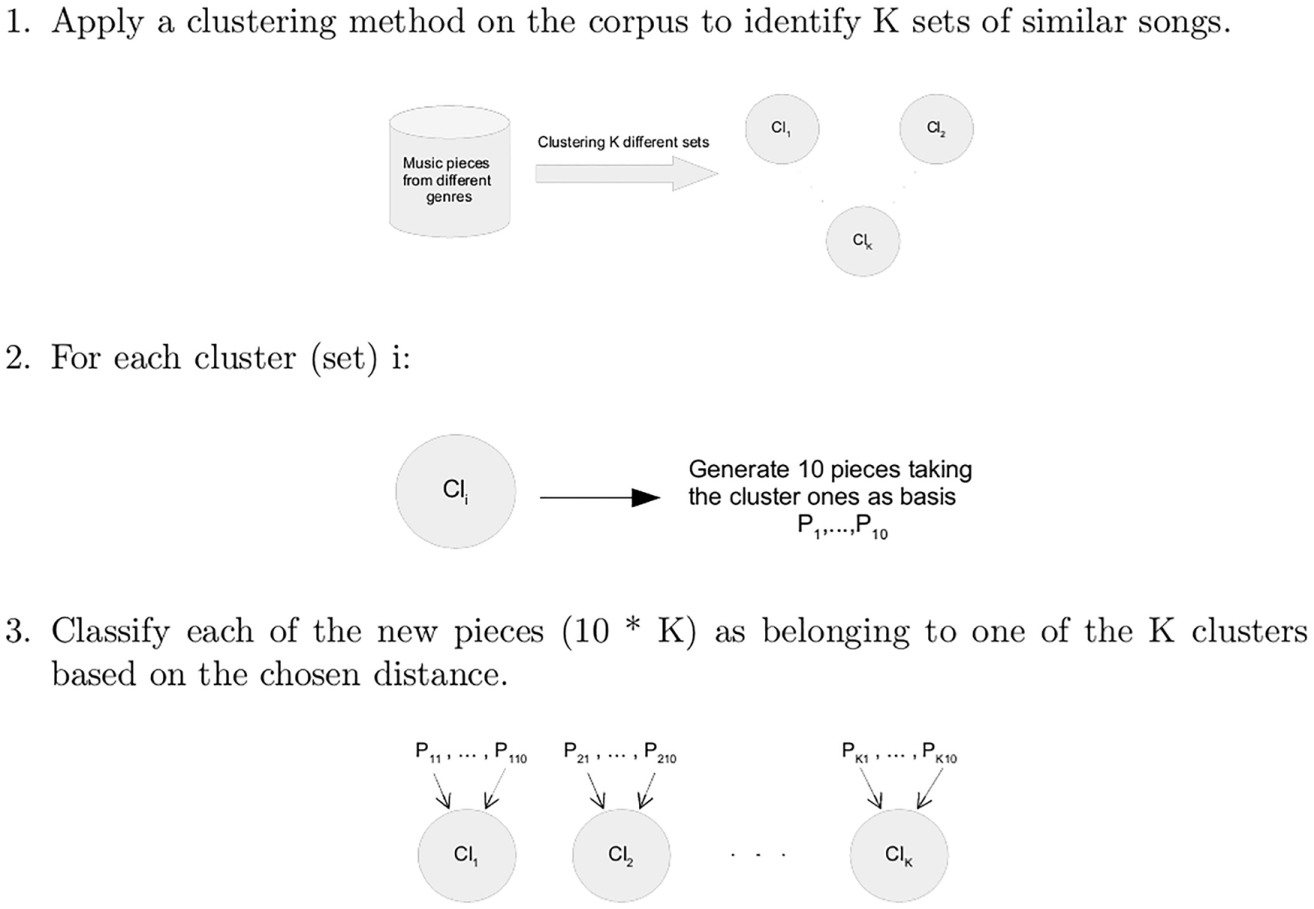
Method. Schema of the method presented in this work.

In this paper a three step method is presented to analyze a melody collection and create *K* clusters of similar melodies, use each of the clusters to generate 10 new pieces and classify each of the new generated pieces in one of the clusters. A schema of the process is shown on Fig 1.

### Corpus

In this work a collection of 100 bertso melodies of the corpus *Bertso doinutegia* is used. Bertso doinutegia is a collection of 2382 bertso melodies, created by Joanito Dorronsoro and published for the first time on 1995. It is updated every year by Xenpelar Dokumentazio Zentroa with new melodies that are used in bertso competitions and exhibitions. Entries in the collection are MIDI files which have a melody name, the name or type of the strophe, type of the melody (genre), creator, bertsolari who has used it, name and location of the person who has collected the melody, and year of the collection. Melodies have been manually classified in 17 genres according to their melodic features and the lyrics that are usually related to them.

**Fig 2:**
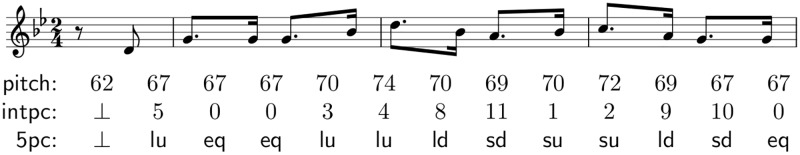
Viewpoint representation. Viewpoint representation of the first two bars of the melody *Abiatu da bere bidean*.

To perform the classification task presented in this work, the melodies in the collection are represented using a viewpoint representation, presented in [37]. A viewpoint *τ* is a function that maps an event sequence *e*_1_, …, *e*_*ℓ*_ to a more abstract derived sequence *τ*(*e*_1_), …, *τ*(*e*_*ℓ*_), comprising elements in the codomain of the function *τ*. Two viewpoints have been selected to represent the pieces in the corpus; pitch class interval (intpc) which computes the shortest distance in pitch class space between two unordered pitch classes (mod 12 interval), and five point contour (5pc) which represent the contour between two consecutive notes. A five point representation is used for contour, where ld and lu records whether a note is approached by a leap of three or more semitones (down or up), sd and su represent a step (smaller than three semitones) approximation and eq represents a unison. Fig 2 shows the viewpoint representation of the first two bars of the melody *Abiatu da bere bidean*, where the pitch class interval and five point contour representations of the notes in the segment can be seen, along with their pitch numbers.

### Matrices

**Fig 3:**
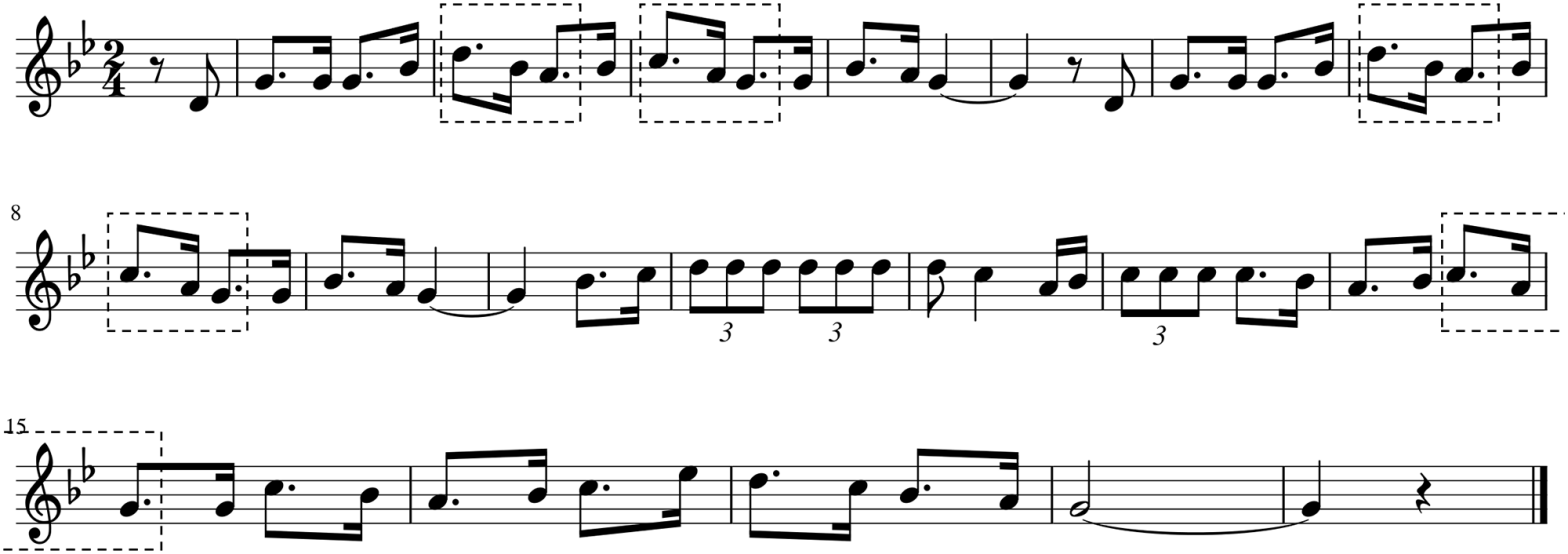
Example score. Score of the melody *Urruti nere menditik* included in the corpus. Contour sequences [ld,sd] are highlighted.

**Fig 4:**
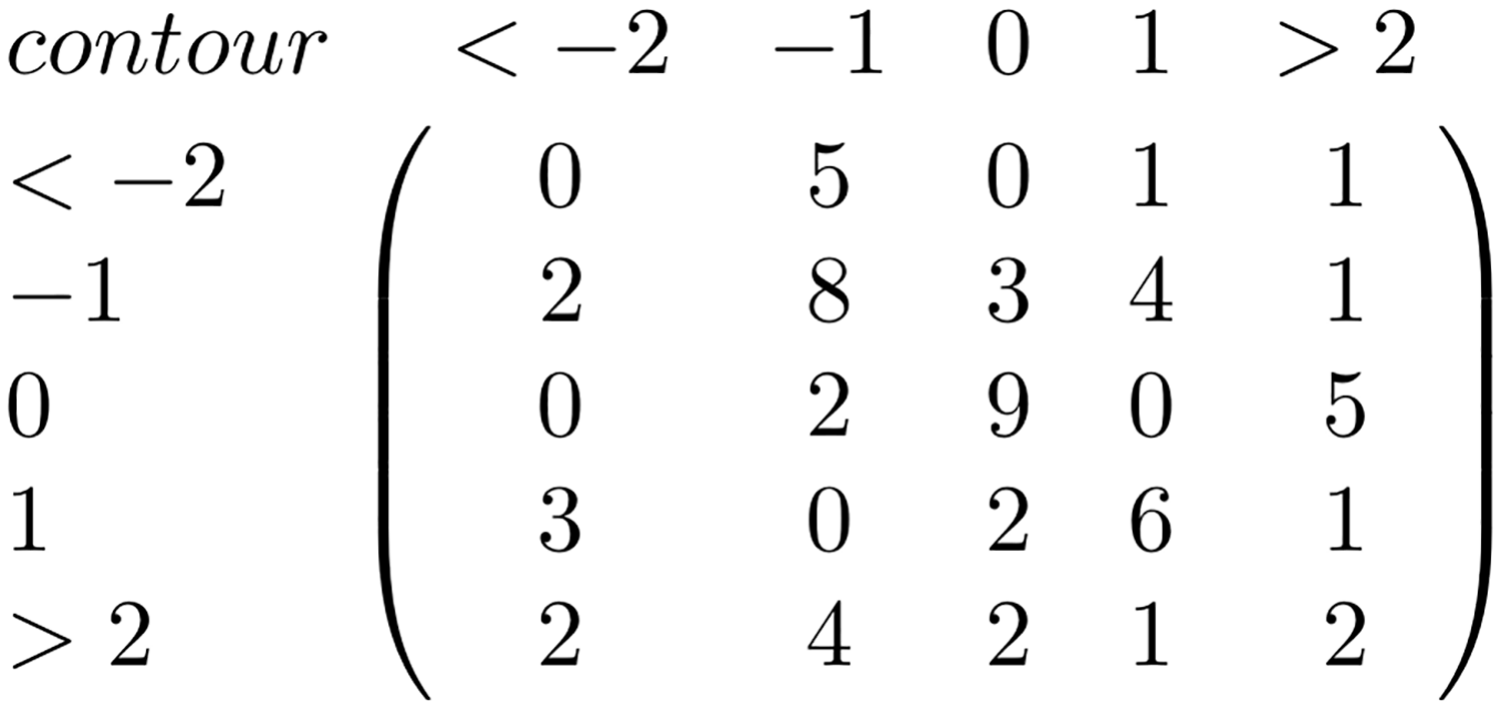
Contour matrix. Example of a contour matrix extracted from the piece *Urruti nere menditik*. Contours ld and lu represent a leap down or up of three or more semitones, contours sd and su represent a step down or up of one or two semitones and contour eq represents unison.

**Fig 5:**
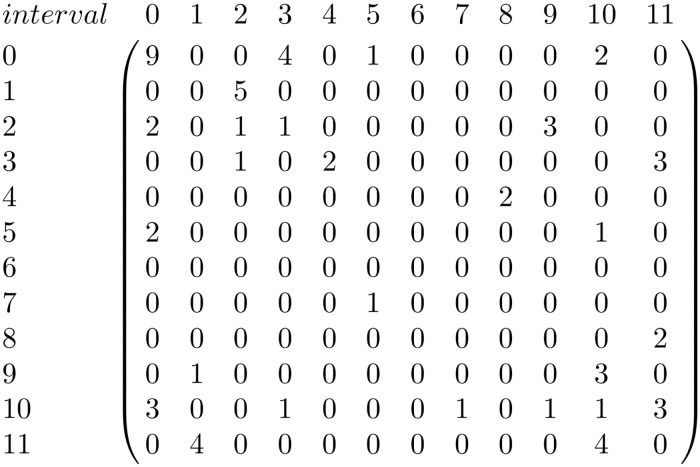
Interval matrix. Example of an interval matrix from the piece *Urruti nere menditik*.

In order to discover similarities between the different pieces in the corpus they are represented using matrices that capture their melodic information. Using the intpc and 5pc viewpoints two matrix types are defined; interval matrices and contour matrices. Interval matrices are 12×12 matrices which count the number of transitions between all the interval pairs that occur in each melody. In order to build them the mod 12 interval between each contiguous note pair is computed. Then, the number of occurrences of each possible interval transition is computed. On the other hand, contour matrices are 5×5 matrices which count the number of transitions between all the contour pairs of each piece. To build the contour matrices the contour transition between each pair of notes is computed and represented using the five point representation presented on Section ‘corpus’. Then, the number of occurrences of each possible contour transition is computed. A contour matrix and an interval matrix are computed for each piece in the corpus. An example of a contour matrix and an interval matrix extracted from the piece in Fig 3 are shown in Figs 4 and 5.

To compute a position in the contour matrix, for example the [ld,sd], the number of times in the piece where a contour leap down (an interval larger than two semitones down) is followed by a contour step down (a step of one or two semitones down) is counted, which in this piece is 5. On Fig 3 these sequences have been highlighted to illustrate better where these sequences can be found on the example score shown.

### Unsupervised classification

With the matrices obtained in the previous step, a method to group together similar songs has been developed through an unsupervised learning process.

In order to discover relationships among the songs, an agglomerative hierarchical clustering algorithm has been used (Sequential Agglomerative Hierarchical Non-overlapping algorithm (SAHN)) [38]. This algorithm starts with a partition where each case is associated to a different cluster, therefore there are so many clusters as different cases. At each subsequent step the algorithm merges two clusters following certain optimization criteria, until all the data belongs to the same cluster. The output of the algorithm is a hierarchy along with the merging steps. Then, if a partition with *N* clusters is wanted, it is necessary to traverse the hierarchy until the right cutting point is found. The criteria to merge two clusters in the building phase is the complete linkage method, where the distance between two clusters is the maximum distance between their individual components.

**Fig 6:**
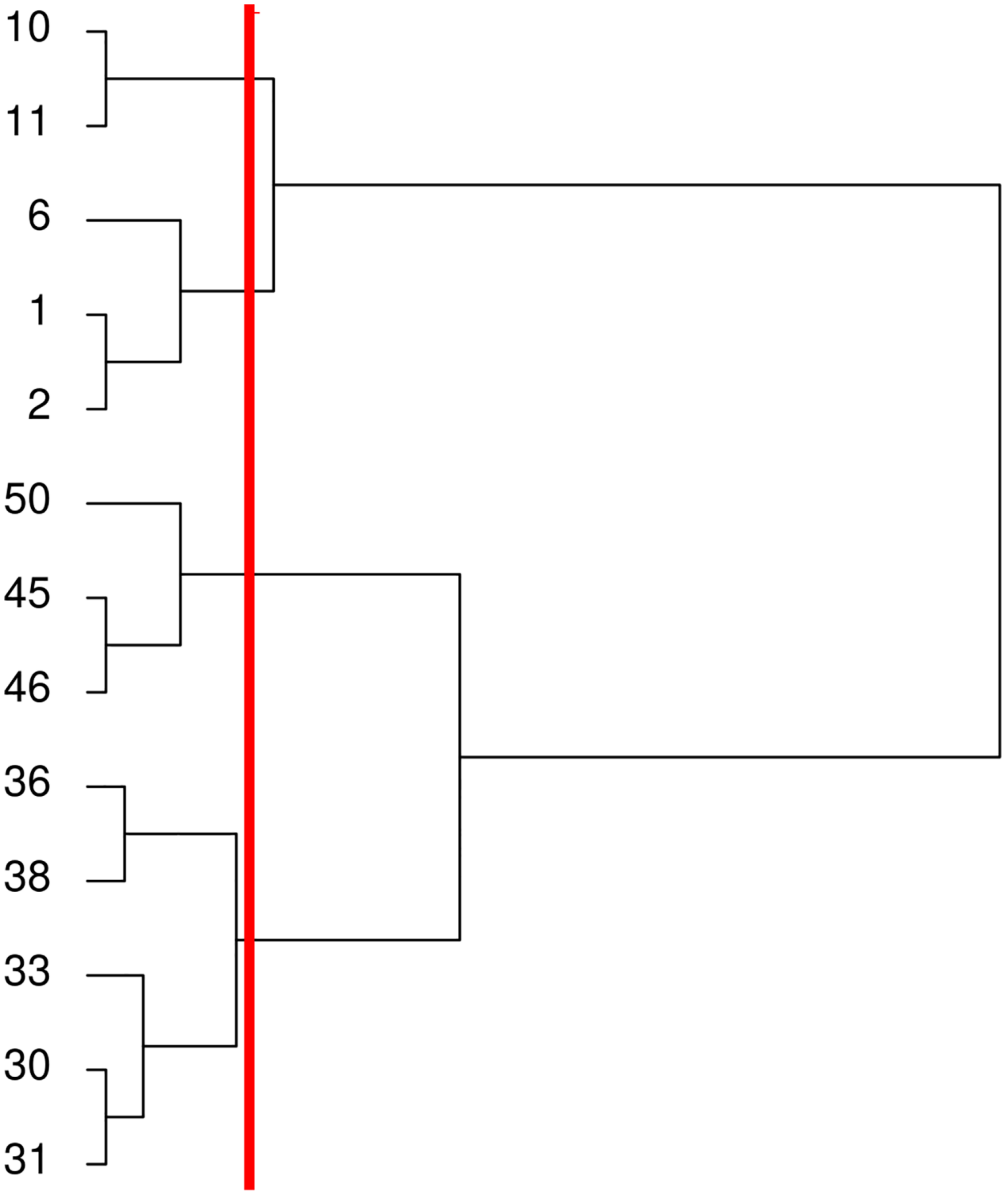
Example dendrogram. Example of a dendrogram created by the SAHN method.

In Fig 6 is shown an example of a dendrogram showing the clusters created after applying the SAHN method to the set of numbers {1,2,6,10,11,30,31,33,36,38,45,46,50}. As it can be seen from the figure, sets of numbers that are very close to each other according to the complete linkage method are grouped together lower in the hierarchy, while the sets that are father apart are grouped in the top. If we are interested in the partition with a given number of clusters, it is necessary to check the level of the dendrogram where such partition is created. For example, the red vertical line of Fig 6 shows the level of the dendrogram where a partition of four clusters is created. These clusters are {10,11}, {1,2,6}, {45,46,50} and {30,31,33,36,38}.

In the research described in this paper matrices representations are used, and therefore suitable distances between matrices are needed. Several distances have been tested. These distances are the following ones:

- The distances induced by the following norms: 1-norm, ∞-norm, Frobenius norm, maximum modulus norm.
- Kullback-Leibler and Jeffrey divergences.
- Earth mover’s, Manhattan and Intersect distances.

In the following paragraphs we will explain them briefly:

#### 1-norm

The 1-norm is computed as the maximum of the sums of the absolute values of the elements of each column. For an M-by-N matrix A, its value is

$$\begin{array}{c} \max_{1\leq j\leq N}\sum_{i=1}^{M}\left|a_{ij}\right|. \end{array}$$

#### ∞-norm

The ∞-norm is computed as the maximum of the sums of the absolute values of the elements of each row. For an M-by-N matrix A, its value is

$$\begin{array}{c} \max_{1\leq i\leq M}\sum_{j=1}^{N}\left|a_{ij}\right|. \end{array}$$

#### Frobenius norm

The Frobenius norm (F-norm) of a matrix, sometimes also called the Euclidean norm, is computed as the square root of the sum of the absolute squares of its elements. For an M-by-N matrix A, its value is

$$\begin{array}{c} \sqrt{\sum_{i=1}^{M}\sum_{j=1}^{N}{\left|a_{ij}\right|}^{2}}. \end{array}$$

#### Maximum modulus norm

The maximum modulus norm (M-norm) of a matrix is computed as the maximum of the absolute values of its elements. For an M-by-N matrix A, its value is

$$\begin{array}{c} \max_{1\leq i,j\leq M,N}\left|a_{ij}\right|. \end{array}$$

#### Kullback-Leibler divergence

The Kullback-Leibler divergence (KL) can be interpreted as the number of additional bits needed to encode instances coming from a distribution *p*(*x*) if coded according with another distribution *q*(*x*). For two M-by-N matrices A and B interpreted as distributions over a two-dimensional grid, its value is

$$\begin{array}{c} \sum_{1\leq i,j\leq M,N}a_{ij}\log\frac{a_{ij}}{b_{ij}}. \end{array}$$

#### Jeffrey divergence

The Jeffrey divergence is a measure that tries to address one of the problems of the Kullback-Leibler divergence, the lack of symmetry. It is defined as

$$\begin{array}{c} D_{KL}\left(A,B\right)+D_{KL}\left(B,A\right). \end{array}$$

#### Earth mover’s distance

The earth mover’s distance (EMD) is a distance between two probability distributions. The name comes from its intuitive interpretation: if the probability distributions are modelled as amounts of material over a surface, the EMD distance is the cost of moving the amounts from one disposition to another. For two M-by-N matrices A and B interpreted as distributions over a two-dimensional grid, its value is

$$\frac{\sum_{1\leq i,j\leq M,N}\sum_{1\leq k,l\leq M,N}f_{ijkl}d_{ijkl}}{\sum_{1\leq i,j\leq M,N}\sum_{1\leq k,l\leq M,N}f_{ijkl}}.$$

where *f*_*ijkl*_ is the flow between *a*_*ij*_ and *b*_*kl*_ that minimizes the total cost, with *d*_*ijkl*_ the distance between the elements *a*_*ij*_ and *b*_*kl*_.

#### Manhattan distance

The Manhattan distance between two M-by-N matrices A and B is defined as

$$\begin{array}{c} \sum_{i=1}^{M}\sum_{j=1}^{N}\left|a_{ij}-b_{ij}\right|. \end{array}$$

#### Intersect distance

The Intersection distance between two M-by-N matrices A and B is defined as

$$\begin{array}{c} \sum_{i=1}^{M}\sum_{j=1}^{N}\min\left(a_{ij},b_{ij}\right). \end{array}$$

These distances or norms are all used in our work; interested readers could refer to [39] to have a better view and further knowledge about distances and their use in Machine Learning.

After applying the SAHN algorithm with the previous matrices distances to the pieces in the corpus, several clusters partitions are created. Those clusters partitions are used to generate new melodies that are intended to be similar to the original pieces.

### Music generation

To generate new melodies a music generation method based on statistical models and a coherence structure is used. The coherence structure of a piece describes which segments are related on a piece, where the relations between segments can be exact repetitions or transpositions. Transposed segments are segments that have the same interval sequence, but different notes. A coherence structure is extracted from a template piece and is then used to guide the generation process in order to get new coherent melodies. As a result of the process pieces that have the same coherence structure of the template, but different melodic content, are created.

#### Coherence structure

In order to extract the coherence structure of a melody a manual or automatic segmentation is performed to identify the segments that are related through the piece. Many related segments may exist within a piece, but the most meaningful ones are retained, manually creating a structure of segments that do not overlap. The extracted structure is then used as a guide on the generation of new musical information, which segments in the new melody must be repeated or transposed.

**Fig 7:**
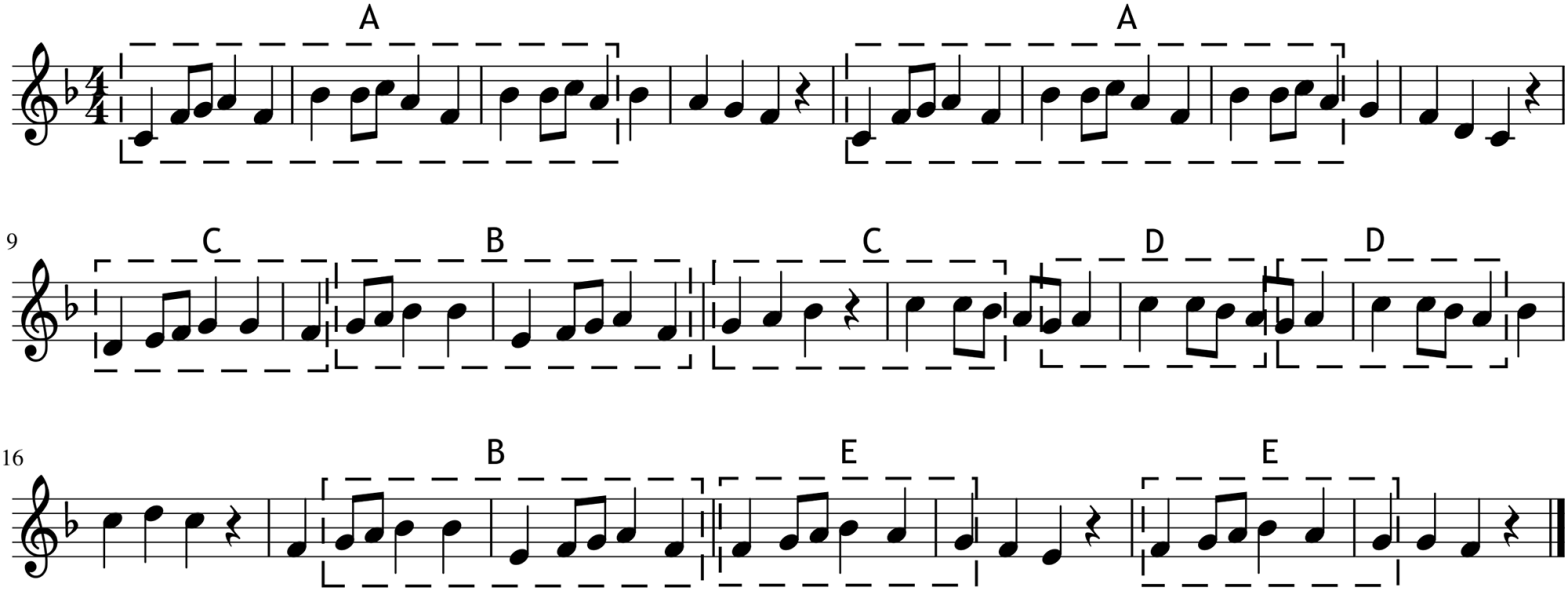
Segmentation example. Example of a segmentation performed on the template piece *Abiatu da bere bidean* used in this work. All the different segments are labelled from A to E, where A, B, D and E are repetition segments and C is a transposed segment.

Fig 7 shows a segmentation for one of the pieces used as templates in the generation, where several segments have been highlighted. Segments A, B, D and E are repetition segments, they occur twice unaltered within the piece, and segment C is a transposition segment.

In the generation process the defined coherence structure will be used as a constraint, to assure that the generated melodies respect the coherence of the template piece.

#### Statistical models

A statistical model is built from each of the clusters identified in the previous step of the presented method. Once it is built, it is used to measure the probabilities of the generated melodies, using the single viewpoint model described in [40] and presented in the equation below. Letting *v*_*i*_ = *τ*(*e*_*i*_|*v*_*i*_, *e*_*i*−1_) be the feature *τ* of event *e*_*i*_ in the context of its preceding event *e*_*i*−1_, the probability of the piece is computed as:

$$\begin{array}{c} \begin{array}{c} P\left(e\right)=\prod_{i=1}^{\ell }P\left(v_{i}\right)\times P\left(e_{i}|v_{i},e_{i-1}\right).\\ P\left(e_{i}|v_{i},e_{i-1}\right)={\left|\left\{x:\tau \left(x|e_{i-1}\right)=v_{i}\right\}\right|}^{-1}. \end{array} \end{array}$$ (1)

On trained and validated models, sequences having high probability are assumed to retain more aspects of the music style under consideration than sequences with low probability, therefore, they are considered better melodies.

#### Sampling

**Fig 8:**
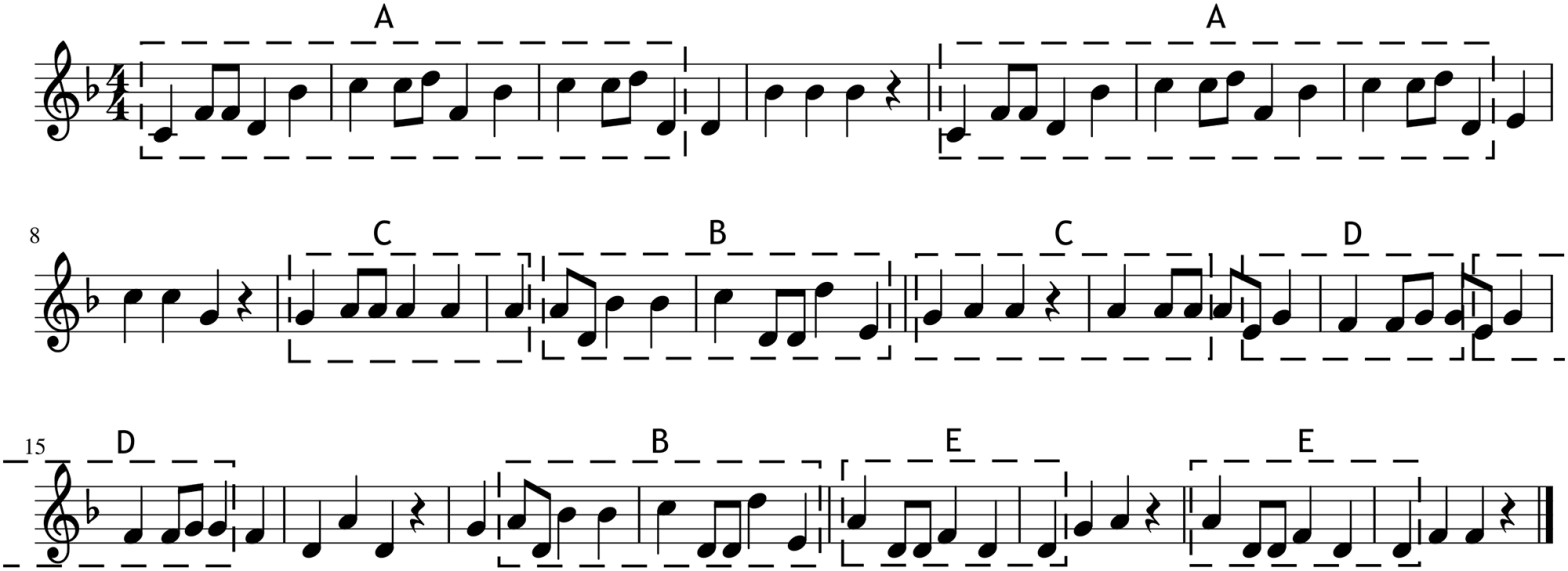
Sampling starting point. Example of a starting point for the stochastic hill climbing method.

The sampling process consists on generating new melodic information that respects the coherence structure extracted from the template piece with a high probability according to the statistical model created from the corpus. For sampling a *stochastic hill climbing* optimization method is used, which is iterated 10^4^ times. This method takes a new piece as a starting point, which respect the coherence structure extracted from the template piece and which has random notes sampled into the different segments of the structure. To create it a left to right sampling is used, which samples random notes into each position of the piece, including the positions that are not part of any segment of the coherence structure. Every time a whole segment is sampled all the other occurrences of the segment are also sampled. In Fig 8 an example of a piece generated as a starting point for this method is shown. The highlighted segments show that the coherence of the template piece is respected, but the notes within the segments are randomly selected. It can be seen that the melody is not smooth, it has many big leaps between the notes, which is not very common in the melodies used in the corpus, making its probability low.

**Fig 9:**
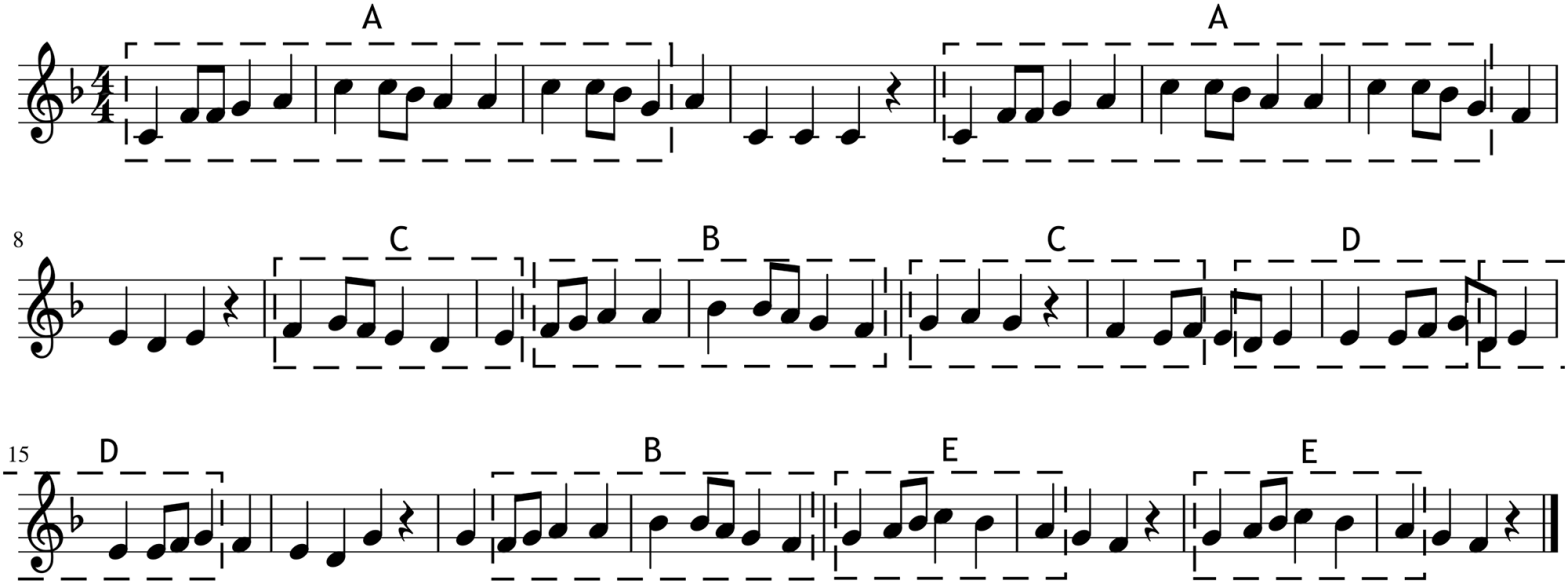
Generation example. Example of a melody generated using the coherence structure of the melody *Abiatu da bere bidean*, shown in Fig 7.

In order to improve the generated piece the method modifies it iteratively, where in each iteration a random location in the piece is chosen and a random note from the vocabulary of the template piece is substituted into that position, producing a new piece with an updated probability, computed using the Eq 1. If the new probability is higher than the last saved one the change is conserved. To conserve the coherence structure of the original template every time a position that is covered by a segment is changed all the other occurrences of that segment are also changed. Fig 9 shows an example generation guided by the coherence structure of the template piece shown in Fig 7. It can be seen that even if the melodies are different they share the repetition structure, which should endow the generations with coherence.

## Experimental setup

A set of 100 random pieces of the corpus described in Section ‘corpus’ used to extract a representation of pitch class interval and five point contour viewpoints of each piece, from which the contour and interval matrices of each melody are computed. These matrices are then used to perform an unsupervised classification and group similar songs into clusters. These clusters are then used to build statistical models that are used in the automatic music generation process.

A first experiment with the melody named *Abiatu da bere bidean*, which is part of the corpus, but is not part of the 100 piece set, is used to extract the coherence structure that guides the generation, along with the statistical models computed from the clusters identified in the classification process. 10 different generations have been made for each cluster, and they have been represented as contour and interval matrices to be classified in the next step. Three extra experiments have been performed with three more melodies randomly chosen from the corpus.

## Experimental results

As commented in the previous section, two types of matrices have been obtained for each melody, and both have been used to test the proposed approach.

### Contour

Obtained classification accuracies are shown in Table 1. As it can be appreciated, obtained results are very different regarding the used distance and the number of cluster selected. It can be inferred, indeed, that there is a distance, EMD, which out-stands clearly from the other when a low number of clusters is used. As a matter of fact, the best results are obtained using this EMD distance for cluster numbers 2 and 4; concerning to other number of clusters, normalized distances appear to be the best choice, being M-norm which obtains the best mean among all. It is worth remarking the result obtained by 1-norm distance when six clusters are used: it obtains by far the best result among all the distances used (0.583).

**Table 1:** Contour: Obtained accuracies by distance type and cluster number.

Cluster Num	2	3	4	5	6	Mean
1-norm	0.500	0.417	**0.500**	0.350	**0.583**	0.470
∞-norm	0.500	0.417	0.250	0.250	0.208	0.325
M-norm	0.750	**0.750**	0.438	**0.550**	0.417	**0.581**
F-norm	0.625	0.417	0.375	0.100	0.250	0.353
EMD	**0.875**	0.667	**0.500**	0.450	0.333	0.565
Jeffrey	0.500	0.333	0.250	0.250	0.167	0.300
Manhattan	0.500	0.417	0.250	0.250	0.167	0.317
Intersect	0.375	0.333	0.250	0.200	0.125	0.257
KL	0.375	0.333	0.313	0.450	0.417	0.378

### Interval

The same experiment has been repeated, using Interval type matrices, and the obtained accuracy results have been presented in Table 2. In this case, EMD distance out-stands as the best one in the performed experiments; best results are obtained using this distance for 3 to 6 clusters, and the best mean is obtained with this distance as well. Remarkable result of Manhattan distance for two clusters (0.875), which makes it candidate for low cluster situations; it obtains the second best mean among all distances.

**Table 2:** Interval: Obtained accuracies by distance type and cluster number.

Cluster Num	2	3	4	5	6	Mean
1-norm	0.500	0.333	0.375	**0.400**	0.292	0.380
∞-norm	0.750	0.333	0.313	0.250	0.167	0.363
M-norm	0.625	0.333	0.250	0.200	0.250	0.332
F-norm	0.500	0.167	0.250	0.200	0.333	0.290
EMD	0.500	**0.667**	**0.625**	**0.400**	**0.542**	0.547
Jeffrey	0.750	0.500	0.313	0.150	0.083	0.359
Manhattan	**0.875**	0.333	0.250	**0.400**	0.375	0.447
Intersect	0.500	**0.667**	0.188	0.150	0.125	0.326
KL	0.625	0.333	0.250	0.200	0.083	0.298

### Extra experiments

In order to provide a better overview of the proposed approach, a set of extra experiments have been set up; 3 pieces have been randomly selected for the corpus. These new three melodies are *Aita semeak tabernan daude I* (which from now on will be identified with the melody ID 1360), *Gure herriko bikariuak* (melody ID 1476) and *Zazpi ahizparen gai den oihala I* (melody ID 1599). The approach presented in this paper has been applied taking as template piece each melody of the new experiment set.

Tables 3 and 4 show the obtained results for the first piece (melody ID 1360) for contour and interval representation respectively. As it can be seen, the same result is obtained for the 2 clusters scenario, but the results differ between both representations in the remaining cluster numbers considered. Interval representation is slightly better, although the best distance mean is obtained by M-norm in the Contour case. Different distances obtain the best result for different cluster numbers, which indicates that the appropriate one should be carefully selected for each considered case.

**Table 3:** Contour: Obtained accuracies by distance type and cluster number (melody ID 1360).

Cluster Num	2	3	4	5	6	Mean
1-norm	0.500	0.333	0.250	**0.380**	0.283	0.349
∞-norm	0.500	0.333	0.450	0.360	**0.333**	0.395
M-norm	**0.750**	**0.500**	**0.475**	0.320	0.183	**0.446**
F-norm	0.500	0.333	0.300	0.140	0.183	0.291
EMD	0.500	0.367	0.300	0.240	0.267	0.335
Jeffrey	0.400	0.333	0.250	0.160	0.017	0.232
Manhattan	0.500	0.333	0.300	0.280	0.183	0.319
Intersect	0.250	0.300	0.250	0.200	0.183	0.237
KL	0.550	0.400	0.275	0.320	0.217	0.352

**Table 4:** Interval: Obtained accuracies by distance type and cluster number (melody ID 1360).

Cluster Num	2	3	4	5	6	Mean
1-norm	0.500	0.333	0.350	**0.360**	0.317	0.372
∞-norm	0.650	0.333	0.250	0.300	0.183	0.343
M-norm	0.500	0.333	0.275	0.200	0.267	0.315
F-norm	0.500	0.333	0.250	0.200	0.267	0.310
EMD	0.500	0.333	**0.475**	0.200	0.267	0.355
Jeffrey	0.650	0.300	0.325	0.320	0.133	0.346
Manhattan	0.700	0.367	0.425	0.260	0.317	**0.414**
Intersect	0.500	**0.667**	0.250	0.240	**0.350**	0.401
KL	**0.750**	0.233	0.075	0.200	0.067	0.265

Regarding the second piece (melody ID 1476), obtained results are shown in Tables 5 (contour) and 6 (interval). In this case, interval representation is the best one, being the best mean accuracy obtained using the EMD distance. When the number of clusters is 2 or 3, the M-norm distance is the one which obtains better results.

**Table 5:** Contour: Obtained accuracies by distance type and cluster number (melody ID 1476).

Cluster Num	2	3	4	5	6	Mean
1-norm	0.500	0.333	0.300	0.400	0.333	0.373
∞-norm	0.500	0.333	0.325	0.300	0.233	0.338
M-norm	0.500	0.600	**0.450**	0.380	**0.433**	**0.473**
F-norm	0.550	0.333	0.275	0.260	0.183	0.320
EMD	**0.650**	**0.533**	0.425	0.420	0.200	0.446
Jeffrey	0.500	0.400	0.275	0.100	0.233	0.302
Manhattan	0.500	0.333	0.275	0.220	0.300	0.326
Intersect	0.200	0.033	0.250	0.140	0.150	0.155
KL	0.550	0.400	0.300	**0.440**	0.133	0.365

**Table 6:** Interval: Obtained accuracies by distance type and cluster number (melody ID 1476).

Cluster Num	2	3	4	5	6	Mean
1-norm	0.650	0.400	0.500	0.280	0.300	0.426
∞-norm	0.600	0.433	0.325	0.160	0.183	0.340
M-norm	**0.800**	**0.733**	0.450	0.160	0.317	0.492
F-norm	0.500	0.333	0.300	0.260	0.217	0.322
EMD	0.750	0.433	**0.550**	**0.440**	**0.350**	**0.505**
Jeffrey	0.650	0.067	0.075	0.160	0.050	0.200
Manhattan	0.500	0.467	0.375	0.280	0.233	0.371
Intersect	0.500	0.400	0.350	0.140	0.133	0.305
KL	0.450	0.400	0.200	0.060	0.233	0.269

For the third selected musical piece (melody ID 1599) the obtained results are shown in Tables 7 and 8 for contour and interval representation respectively. Once again, interval is the best representation, and the results differ depending on the number of clusters used. The best mean is obtained by M-norm distance for contour representation.

**Table 7:** Contour: Obtained accuracies by distance type and cluster number (melody ID 1599).

Cluster Num	2	3	4	5	6	Mean
1-norm	0.500	0.333	**0.500**	**0.500**	**0.500**	0.467
∞-norm	0.500	0.333	0.250	0.200	0.200	0.297
M-norm	0.700	**0.800**	**0.500**	0.440	0.417	**0.571**
F-norm	0.600	0.500	0.400	0.200	0.333	0.407
EMD	**0.750**	0.667	**0.500**	0.400	0.267	0.517
Jeffrey	0.350	0.500	0.450	0.360	0.033	0.339
Manhattan	0.500	0.567	0.350	0.240	0.400	0.411
Intersect	0.650	0.367	0.275	0.160	0.133	0.317
KL	0.200	0.367	0.350	0.220	0.233	0.274

**Table 8:** Interval: Obtained accuracies by distance type and cluster number (melody ID 1599).

Cluster Num	2	3	4	5	6	Mean
1-norm	0.700	0.467	0.675	**0.500**	0.400	**0.548**
∞-norm	**0.900**	0.333	0.250	0.340	0.267	0.418
M-norm	0.750	**0.600**	0.400	0.320	0.300	0.474
F-norm	0.500	0.333	0.250	0.200	0.250	0.307
EMD	0.550	0.433	**0.725**	0.400	0.450	0.512
Jeffrey	0.500	0.267	0.125	0.140	0.167	0.240
Manhattan	0.500	0.500	0.350	0.360	**0.483**	0.439
Intersect	0.500	0.533	0.175	0.160	0.133	0.300
KL	0.600	0.500	0.275	0.120	0.067	0.312

It is worth mentioning that the results obtained in the extra experiments do not differ with the ones shown in Tables 1 and 2 which indicates that the proposed approach gives an accurate way to classify different songs once the model has been trained using an appropriate subset of representative melodies.

## Conclusions and future works

In this paper an investigation of the classification of automatically generated melodies is performed; the main idea that grouping close related known pieces in different sets –or clusters–, and afterwards generating new melodies in an automatic way, which are somehow “inspired” in each set. The new melodies are supposed to be classified to this set, using the same distance used to identify the clusters.

Although obtained results could be seen as not so good for other kind of data –we do not expect a medical research giving us a 66% of suffering a disease, or a industrial task telling us that certain piece is among tolerance-threshold on a 56% probability– it has to be remarked the artistic environment the performed experiment have been carried out, in an area which is no deterministic, and in genres that could be confused among each other.

Nevertheless, obtained results indicate the appropriateness of the whole process: results over 0.5 can be considered encouraging, especially when the cluster number is 4 or more. Some extra experiments have been performed using three different songs as template, and using the previously obtained clustering as classification model. Obtained results are similar to the previous ones, which indicates the soundness of the proposed approach.

As future work a deeper analysis is envisaged, and a combination of both representations (contour and interval) in order to obtain a better idea of the genre divisions obtained by the clustering process. Another open line remain in the use of different distances to classify the new generated melodies and to divide the existing songs in different clusters. On the music generation topic the rhythm generation and the use of harmonic information to generate melodies are lines that should also be studied in the future.
